# Reduced Fronto-Temporal and Limbic Connectivity in the 22q11.2 Deletion Syndrome: Vulnerability Markers for Developing Schizophrenia?

**DOI:** 10.1371/journal.pone.0058429

**Published:** 2013-03-22

**Authors:** Marie-Christine Ottet, Marie Schaer, Leila Cammoun, Maude Schneider, Martin Debbané, Jean-Philippe Thiran, Stephan Eliez

**Affiliations:** 1 Office Médico-Pédagogique (OMP), University of Geneva School of Medicine, Geneva, Geneva, Switzerland; 2 Signal Processing Laboratory (LTS5), Swiss Federal Institute of Technology (EPFL), Lausanne, Vaud, Switzerland; 3 Adolescence Clinical Psychology Research Unit, University of Geneva, Geneva, Geneva, Switzerland; 4 Department of Genetic Medicine and Development, University of Geneva School of Medicine, Geneva, Geneva, Switzerland; Beijing Normal University, China

## Abstract

The 22q11.2 deletion syndrome (22q11DS) is a widely recognized genetic model allowing the study of neuroanatomical biomarkers that underlie the risk for developing schizophrenia. Recent advances in magnetic resonance image analyses enable the examination of structural connectivity integrity, scarcely used in the 22q11DS field. This framework potentially provides evidence for the disconnectivity hypothesis of schizophrenia in this high-risk population. In the present study, we quantify the whole brain white matter connections in 22q11DS using deterministic tractography. Diffusion Tensor Imaging was acquired in 30 affected patients and 30 age- and gender-matched healthy participants. The Human Connectome technique was applied to register white matter streamlines with cortical anatomy. The number of fibers (streamlines) was used as a measure of connectivity for comparison between groups at the global, lobar and regional level. All statistics were corrected for age and gender. Results showed a 10% reduction of the total number of fibers in patients compared to controls. After correcting for this global reduction, preserved connectivity was found within the right frontal and right parietal lobes. The relative increase in the number of fibers was located mainly in the right hemisphere. Conversely, an excessive reduction of connectivity was observed within and between limbic structures. Finally, a disproportionate reduction was shown at the level of fibers connecting the left fronto-temporal regions. We could therefore speculate that the observed disruption to fronto-temporal connectivity in individuals at risk of schizophrenia implies that fronto-temporal disconnectivity, frequently implicated in the pathogenesis of schizophrenia, could precede the onset of symptoms and, as such, constitutes a biomarker of the vulnerability to develop psychosis. On the contrary, connectivity alterations in the limbic lobe play a role in a wide range of psychiatric disorders and therefore seem to be less specific in defining schizophrenia.

## Introduction

Neurogenetic syndromes offer a unique framework to study the interplay between genes, brain and behavior [1]. Among neurogenetic conditions, 22q11.2 deletion syndrome (22q11DS), also known as velo-cardio–facial syndrome, is widely recognized as a genetic model for schizophrenia [2], [3]. Indeed, patients affected by 22q11DS show a 30% prevalence rate for developing schizophrenia [4], but 75% present milder psychotic symptoms [5]. Therefore, neuroanatomical measurements in 22q11DS may reveal specific neurodevelopmental pathways [1] and endophenotypic biomarkers for schizophrenia [6].

Nowadays, there is strong evidence that neural dysconnection, namely an abnormal functional integration of brain physiological processes, contributes to symptoms of schizophrenia [7], [8]. As discussed in [7], neural dysconnection may be related either to impairments in synaptic plasticity, or to altered anatomical (long-range) connectivity, or to both processes. Impaired synaptic plasticity in schizophrenia has been suggested using electrophysiological techniques [9] or based on neuropathological examinations [10]. Concerning long-range connectivity, Diffusion Tensor Imaging (DTI) represents a unique opportunity to assess *in vivo* the wiring of white matter connections in patients affected with schizophrenia. An increasing number of DTI studies in patients with schizophrenia are being published and have highlighted aberrant fiber density and organization, most frequently in the prefrontal and temporal regions (reviewed in [11] and [12]). The extent to which abnormal connectivity precedes and predicts the risk of subsequent schizophrenia remains however unclear. Indeed DTI studies in patients at ultra-high risk for psychosis have shown rather inconsistent results, which could be explained by the great heterogeneity of these patients [13].

Several studies have employed DTI to analyze brain connectivity in the 22q11DS, a population with a homogenous risk for schizophrenia. The first DTI studies in 22q11DS [14], [15], [16], [17] used Fractional Anisotropy (FA), which is a measure of the global white matter integrity including their myelination status. The most commonly reported findings were a reduction of FA in the parietal and frontal regions [15], [17]. However, Fractional Anisotropy analyses do not address the question of whether the trajectories of the bundles (white matter fibers) are similar between the group of patients with 22q11DS and the group of controls. For that purpose, the novel three-dimensional tractography technique [18], provides an unprecedented insight on the organization of the white matter pathways and offers the possibility to explore which particular bundles are affected in the 22q11DS**.** Only two tractography studies have been published to date concerning the 22q11DS, both focusing on the study of the corpus callosum to validate novel image processing techniques [19], [20]. To the best of our knowledge, tractography has never been applied to quantify the pattern of whole brain connections in patients with 22q11DS compared to control participants.

In the present study, we used the connectome technique [21], [22] in a sample of 30 patients affected with 22q11DS and 30 healthy participants matched for age and gender. This method enables to quantify the brain’s global structural connectivity through the extraction of anatomically organized whole-brain connection matrices. These matrices can then be compared between the groups to identify possible brain connectivity alterations. As previous findings in 22q11DS suggest, we expect to find alterations in and in-between lobes. Therefore, we compared lobar connectivity between the two groups. We firstly expected to observe connectivity differences inside the frontal and the occipital lobe, which are frequently reported in patients with 22q11DS. Secondly we also expected differences in the fronto-temporal connectivity, which are frequently implicated in the pathogenesis of schizophrenia.

## Methods

### Sample

#### 22q11DS group

Thirty participants with 22q11DS were recruited through parent associations in France, Belgium and Switzerland. All participants and their parents were informed about the study and signed a consent. The protocol was approved beforehand by the Institutional Review Board of Geneva University School of Medicine. The 22q11DS group included 13 girls and 17 boys aged between 7 and 25 years old (mean = 14.8±4.0). The 22q11.2 deletion was confirmed using DNA polymorphism analysis based on a Quantitative Fluorescent Polymerase Chain Reaction (QF-PCR) performed on the deleted region. IQ was measured using the Wechsler Intelligence Scale for Children-Third Edition revised [23] and the Wechsler Adult Intelligence Scale-III [24] for adults. The 22q11DS patient’s mean IQ was 70.62±11.8. On the basis of a clinical evaluation three subjects with 22q11DS met the DSM-IV criteria for a psychotic disorder and seven reported having hallucinations. No participants were under antipsychotic medication. Six patients were under treatment for attention deficit and hyperactivity disorder (methylphenidate).

#### Control group

The control group was recruited among primary school children and among the siblings of patients. The 30 healthy control (HC) participants (14 girls and 16 boys) had a mean age of 14.9±3.7. No HC had a past or present history of psychiatric or neurological disorders. The mean IQ of the HC group was 105.23±11.01.

### Image Acquisition

Two cerebral MRIs were acquired for each participant during the same scanning session with a Siemens Trio 3 Tesla scanner. A T1-weighted sequence with a 3D volumetric pulse was collected using the following sequence: TR = 2500 ms, TE = 3 ms, flip angle = 8°, acquisition matrix of 256×256, field of view = 22 cm, slice thickness = 1.1 mm, 192 slices. The second MRI was a Diffusion Tensor Imaging (DTI) with the following parameters: number of directions = 30, b = 1000 s/mm^2^, TR = 8300 ms, TE = 82 ms, flip angle = 90°, acquisition matrix of 128×128, field of view 25.6 cm, slice thickness = 2 mm.

### Image Processing

The Human Connectome [21], [22] is a technique that combines the reconstruction of the cortical anatomy and the representation of the underlying white matter fiber pathways. Using the Flirt rigid transformation tool of FSL-FDT software [25], [26] we correct the effect of head motion and distortion of eddy currents through an affine alignment of all the weighted diffusion images onto the b0 image, then we register the T1-weighted image on the set of diffusion images. The registered T1-weighted image and the aligned diffusion images are processed separately, producing on one-side accurate mesh models of the cortical surfaces, and on the other side streamlines representing the white matter bundles**.** In the present study, the diffusion images used were Diffusion Tensor Imaging (DTI). Even though the Human Connectome was primarily developed for the use of Diffusion Spectrum Imaging (DSI), the freely available Human Connectome software (connectomics.org) now provides the possibility to reconstruct the streamlines based on either DTI or DSI.

The reconstructions of the cortical surfaces are obtained from the T1-weighted image using the FreeSurfer software (http://surfer.nmr.mgh.harvard.edu). Semi-automated processing allows the reconstruction of accurate three-dimensional mesh models [27], [28] and subcortical regions [29]. The cortical surfaces are subdivided into 66 gyral cortical regions using a validated atlas-based segmentation [30]. FreeSurfer surface reconstruction algorithms have been previously validated against manual delineation on MR images [31] and postmortem brains [32].

To obtain the white matter bundles, the DTI images are processed with the Diffusion Toolkit software (http://trackvis.org/dtk/) using the streamline algorithm [33]. For each voxel of the white matter volume, the signal is combined from the 30 directions to create an ellipsoid diffusion tensor. Then four streamlines are initiated at each voxel of the white matter mask created by Freesurfer and grow voxel by voxel in both directions of the diffusion tensor. The streamline growth process finishes when both ends reach the grey matter mask or when streamlines criteria are reached (max angle 60°, min length 3 mm, max length 1000 mm). Only the curves, called streamlines or fibers, that have both ends finishing at the grey matter mask are retained for the matrix creation. These fibers are estimates of the real white matter axonal bundle trajectories [18], [34], [35].

### Construction of the Connection Matrix

As a result of the procedure described above, 33 cortical regions of interest (ROI) per hemisphere and several thousand white matter fibers were obtained for each participant. A connection matrix is then constructed by grouping each fiber connecting a pair of ROI *i* and *j* into a bundle *B(i,j)*. The value of the connection matrix cell *M(i,j)* is the connection density between the corresponding pair of ROIs, defined as follows:
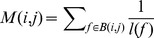
where *l*(*f*) is the length of fiber *f* along its trajectory. The correction term *l(f)* in the denominator is needed to eliminate the linear bias towards longer fibers introduced by the tractography algorithm, which uses each voxel in the white matter mask as a seed point.

The fibers with the lowest FA value are amongst the shortest fibers and their variability is too broad to be considered valid [36]. To overcome this issue, we subtracted these inconsistent fibers using the following approach: 1) an FA matrix was created for each control participant averaging the FA of the fibers connecting each region; 2) the FA matrix of each of the 34 healthy participants was normalized to correct for inter-individual FA differences; 3) the individual matrices were averaged to create a mean FA matrix for the control group. The distribution of these average FA values was explored and revealed a bi-modal distribution, with a narrow distribution in low normalized FA values (0.05 and 0.3) and another in higher FA values (0.3 to 1). After calculating the distribution’s quantiles, we successively removed the cases of the matrix that had a lower FA value than the quantile until we reached a case of the matrix that was not situated in the diagonal (i.e. not a short fiber). Using this technique, we stopped at the 3rd quantile corresponding to a normalized FA below 0.2731. The cases of the connection matrix that showed a mean FA lower than this 3% were excluded from the group comparison test.

### Statistical Analyses

#### Global analyses

We used an ANCOVA to measure the difference in the total number of fibers between the patients and the control group using the SPSS software (http://www.spss.com/). All statistical analyses were controlled for age and gender.

#### Connectivity analyses at the lobar level

The two-step approach used in this study has been chosen for two reasons. First, our sample of 30 individuals with 22q11DS compared to 30 controls was not sufficient to stand the correction for multiple comparisons on a matrix size of 70×70. Most importantly, in 22q11DS literature many findings on lobar resolution have been found using different imaging technique and our concern was to integrate and compare our findings to previous studies. For this purpose, we created a connectivity matrix for each subject, regrouping the 66 cortical and 4 subcortical areas regions into 5 groups representing 5 “lobes” in each hemisphere. Four conventional lobes were defined as the frontal, parietal, occipital and temporal lobes. The fifth “lobe” was defined as the limbic structure, composed of the four parts of the cingulate gyrus, the entorhinal gyrus, the parahippocampal gyrus, the hippocampus and the amygdala.

MANCOVAs were used to measure the differences, between the groups, in the number of fibers connecting the lobes within and between themselves. The analyses were covaried for age, gender and the total number of fibers.

#### Post-hoc analyses at the regional level

When a significant connectivity difference was observed at the lobar level, we then looked at the number of fibers in the cortical parcels composing the relevant lobes. The parcel corresponding to the frontal and temporal poles, as well as the bank of the superior temporal sulcus, were not included in the analyses, as these regions showed poor consistency in the validation article [30]. These regional analyses used MANCOVAs to measure the difference in the number of fibers contained in cortical parcels between groups, covarying for age, gender and total number of fibers.

### Effect of Age

Finally, we explored the effect of age on the white matter parameters. For each participant, the total volume of white matter was calculated from the number of voxels contained in the white matter mask. Mean Fractional Anisotropy of the white matter was then measured for each subject. Then three linear regression analyses were performed between age and 1) the mean fractional anisotropy, 2) the total volume of white matter, 3) the total number of fibers.

## Results

In this section, we will only describe significant findings; p-values are reported in Table 1. As detailed in the *Statistical Analyses* section, all analyses were controlled for the covariation of age and gender. The results presented below always refer to the 22q11DS in comparison to the control group.

**Table 1:** Percentage of absolute and relative difference in the number of fibers inter- and intra-lobe between 22q11DS and the control group and their significance.

Anatomical Structure	Absolute difference (%)	Relative difference (%)	F [1], [58]	P
**Total number of fibers**	90%	/	10.309	0.002
**Right Hemisphere**				
Frontal lobe	99.2%	+9.2%	17.442	<0.001**
Parieto-occipital connections	112.9%	+22.9%	7.148	0.010
Parietal lobe	93.1%	+3.1%	5.826	0.019
Limbic structure	83.5%	−6.5%	6.152	0.016
**Left Hemisphere**				
Fronto-temporal connections	56.1%	−33.9%	11.933	<0.001**
Parieto-limbic connections	79.7%	−10.3%	4.599	0.036
Limbic structure	84.3%	−5.7%	7.065	0.01
Occipital lobe	72%	−18%	10.01	0.003*
**Inter-Hemisphere**				
Inter limbic connections	79.4%	−10.6%	6.363	0.015*
**Right parcels**				
Paracentral	116.6%	+26.6%	18.569	<0.001**
Medial-orbito-frontal	80.7%	−9.3%	12.595	0.001*
Rostral anterior cingulate	63.3%	−26.7%	9.722	0.003*
Lateral-orbito-frontal	112.4%	+22.4%	7.494	0.008*
Inferior parietal	96.2%	+6.2%	6.690	0.012
Rostral-middle-frontal	97.1%	+7.1%	6.306	0.015*
Pars orbitalis	103.7%	+13.7%	6.167	0.016*
**Left parcels**				
Posterior cingulate	77.2%	−12.8%	15.744	<0.001**
Cuneus	73.2%	−16.8%	10.147	0.002*
Parahippocampal	115.6%	+25.6%	17.666	0.008*
Middletemporal	80%	−10%	7.001	0.011
Precuneus	75.6%	−14.4%	6.168	0.016
Isthmuscingulate	82.9%	−7.1%	4.384	0.041

* significant with FDR correction.

** significant with Bonferroni correction.

### Global Results

A significant 10% reduction in the total number of fibers was shown in the 22q11DS group (mean: 43032±4586) in comparison to the control participants (mean: 47423±5739; F_1,58_ = 10.309, p = 0.002).

### Connectivity Results

**Figure 1:**
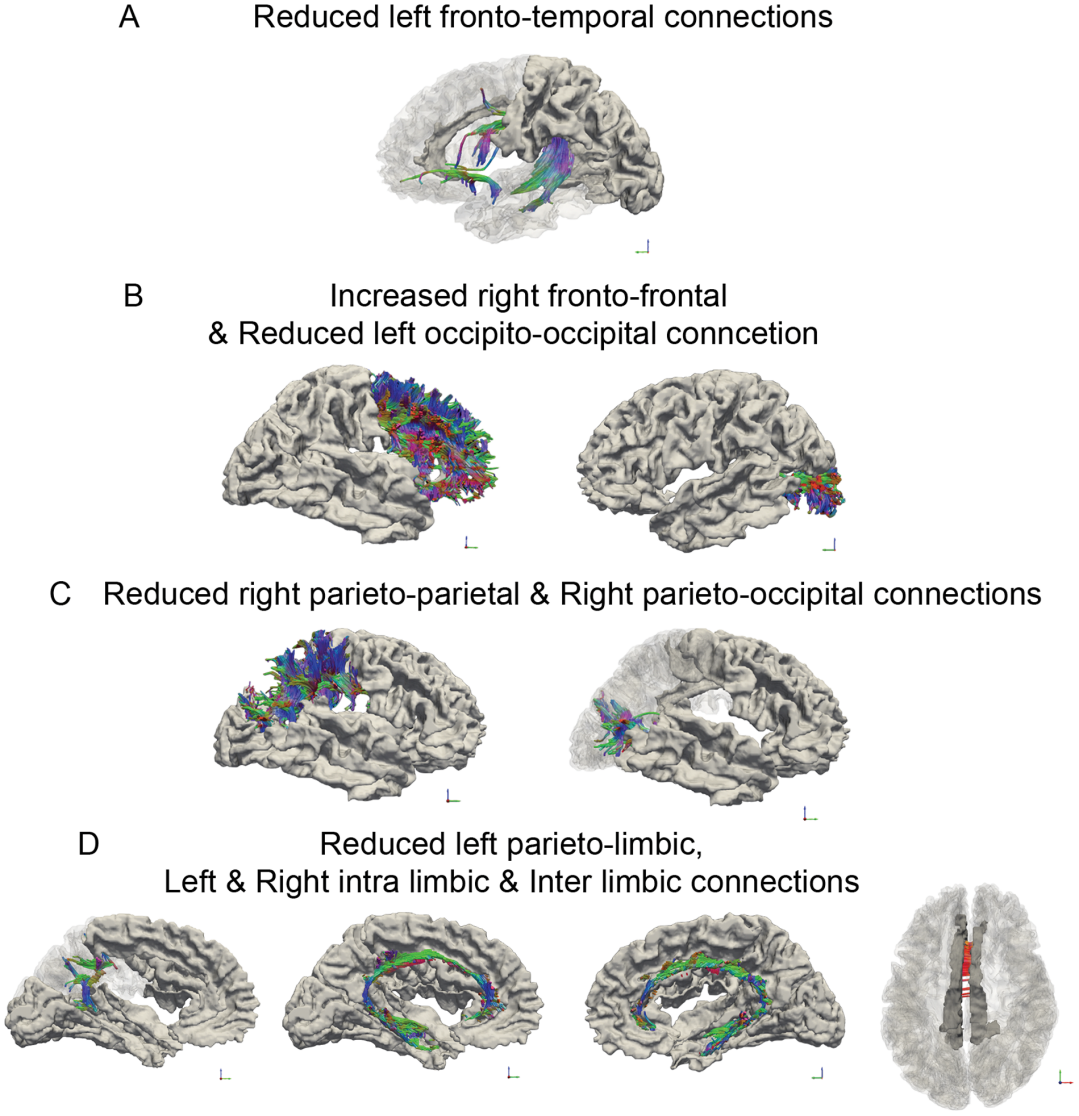
Three-dimensional representations of the virtual fibers with significant differences between patients and controls. Inside the three-dimensional cortical view of each hemisphere in light grey, each segment of the fibers are represented with 3 canonical directional color gradients, green for anterior-posterior axis, blue for bottom-up axis and red for left-right axis. Virtual fibers have been regrouped in “Tube” shape by TrackVis Software (http://trackvis.org/). Part A represents the decrease in the left fronto-temporal connections comprising the arcuate and the uncinate fasciculus (Video S5). Part B represents the increase in the frontal lobe intra connections (Video S1) and the decrease in the left occipital intra connections (Video S8). Part C shows the increase in the right parietal intra connections (Video S2) and parieto-occipital connections (Video S3). Part D illustrates the decrease in bilateral limbic intra and inter connections (Video S4, S7 and S9) and the left parieto-limbic connections (Video S6).

MANCOVAs for each hemisphere and inter-hemisphere connections were corrected for age and gender but also for the 10% reduction observed in 22q11DS for the total number of fibers. Multivariate analyses showed that all intra and inter hemispheric difference between the 22q11DS group and the control group were significant (Wilk’s lambda for right hemisphere p = 0.032, left hemisphere p<0.001 and inter hemisphere p = 0.002). Amongst the involved lobes, we observed preserved areas (i.e. significant increase) as well as disproportionately reduced areas (i.e. significant decrease) in the number of fibers in patients with 22q11DS compared to controls (Figure 1 and Videos S1–S9).

In the right hemisphere, an increase in the number of fibers was observed within the frontal lobe (Video S1), the parietal lobe (Video S2) and in the amount of parieto-occipital connections (Video S3). A significant decrease in the number of fibers was seen within the limbic areas (Video S4). In the left hemisphere, the amount of fibers was significantly decreased in the fronto-temporal (Video S5) and the parieto-limbic connections (Video S6), and within the limbic (Video S7) as well as within the occipital lobe (Video S8). When considering the inter-hemispheric connections, a significant decreased number of fibers connecting the left and the right limbic areas (Video S9) was found. Videos are included as supplementary files.

### Regional Results

Detailed relative and absolute percentages of the significantly different number of fibers in the cortical parcels between groups are also provided in Table 1. In the right frontal lobe, we found an increased number of fibers in the paracentral parcel, the lateral orbito-frontal parcel, the pars orbitalis parcel and the rostral middle frontal parcel. The medial orbito-frontal parcel showed a decrease in the number of fibers in patients compared to controls.

In the right parietal lobe, an increase in the number of fibers was found in the inferior parietal parcel in patients compared to controls. In the limbic structure, the patients showed a decreased number of fibers in the right rostral anterior cingulate parcel, the left posterior cingulate parcel and the left isthmus cingulate but showed an increased number of fibers in the left parahippocampal parcel. In the left occipital lobe, we observed a decrease in the number of fibers in the cuneus parcel for the patients’ group.

### Effect of Age on White Matter Parameters

In both patient and control groups, a significant increase of the total volume of white matter with age was found (22q11DS: R = 0.425, p = 0.010; HC: R = 0.415, p = 0.011). Over the studied age range, the total mean fractional anisotropy grew with age only in the control group (22q11DS: R = 0.225, p = 0.116; HC: R = 0.317, p = 0.044). The regression analysis shows that the total number of fibers was not dependent of age in both groups (22q11DS: R = 0.163, p = 0.194; HC: R = −0.003 p = 0.495).

## Discussion

### Relationship with Previous DTI Studies in 22q11DS

In this study, we found a significant decrease in the 22q11DS group’s brain connectivity. Indeed a 10% decrease in the total number of fibers was observed in patients with 22q11DS compared to healthy participants.

In the 22q11DS, the lobar analyses revealed excessive reductions in white matter fibers in the left hemisphere for the fronto-temporal and the occipito-occipital connections. The limbic connections were excessively reduced, both within each hemisphere and between the inter-hemispheric limbic regions. Contrarily, relative preservation of white matter fibers was seen in the right fronto-frontal, parieto-parietal and parieto-occipital connections (Figure 1).

At the regional level, relative preservation of connectivity was mainly observed in the right hemisphere (frontal and inferior parietal regions) but also in one region of the left hemisphere (parahippocampal). Excessively reduced connectivity was largely observed in both hemispheres, in the right medial frontal regions (medial orbitofrontal and anterior cingulate), the left inferior frontal, middle temporal and medial posterior regions (posterior cingulate, cuneus, precuneus).

To date, five studies have been published using FA to measure connectivity changes in 22q11DS [14], [15], [16], [17], [37]. Among these five studies, two used previously published sample of patients, either improving the image registration [17] or providing new results correlating connectivity with cognitive skills [14]. Increased FA was reported around the splenium of the corpus callosum [15], [16], but it has been suggested that those results represent a registration artifact [17]. Findings in the frontal lobe have been inconsistent: one study observed a bilateral increased FA [17], whereas other studies observed asymmetric findings between the two hemispheres: increased FA in the left frontal lobe [37] and decreased FA in the right frontal lobe [15], [37]. The findings obtained in the parietal lobe also showed some inconsistencies: increased FA bilaterally [17], reduced FA bilaterally [15] and decreased FA in the right post-central area. As FA values are known to increase between childhood and early adulthood [38], these inconsistencies may rely on the different age ranges of the patients (7–14 years [16], [17], 7–22 years [14], [15], adults [37]).

In this study, we also observed an enlargement of white matter volume with age in both groups, which was associated with an increase of the total mean FA in the control group. On the contrary, the number of fibers was not significantly affected by age in any of the diagnosis groups, suggesting that the connectome technique may not be very sensitive to the maturational changes occurring during childhood and adolescence. The reason for this low sensitivity may rely on the maturation process of white matter: the increase of FA is driven by a reduction of the radial diffusivity [39]. Streamline tractography using DTI images ranks the 3 eigenvectors (axial and both radial diffusivity vectors) from the largest to the smallest and uses only the orientation of the first ranked vector. As a result, the tractography constructs 3D fibers following only the orientation of the first vector, and ignores the radial diffusivity that is known to be the most sensitive measure of the maturational process [38].

### Fronto-temporal Disconnectivity as a Vulnerability Factor for Schizophrenia

Given the relatively low sensitivity of our method to dynamic changes occurring from childhood to adulthood, we argue that our results most likely reveal an altered configuration of white matter tracts that is observable at all ages in the syndrome. Part of the abnormal connectivity that we observe in patients with 22q11DS may indeed constitute a vulnerability factor for schizophrenia, already existing years before the onset of the symptoms. For instance, we found evidence of decreased connectivity in the left fronto-temporal tracts in patients with 22q11DS compared to controls. Disrupted integrity of the left fronto-temporal tract (including the arcuate and uncinate fasciculus) has been largely implicated in the pathogenesis of schizophrenia [40]. More specifically, alterations to the integrity of the left arcuate fasciculus have been related to auditory hallucinations [41], [42]. It has been hypothesized that alterations in the connectivity of the Heschl’s gyrus impairs the ability to monitor inner speech leading to confusions between self generated thoughts and external perceptions. Source monitoring impairment is considered a cognitive marker for schizophrenia [43] and has been previously revealed in 22q11DS [44]. Also, we observed decreased connectivity at several levels in the limbic system of patients with 22q11DS (within the limbic system bilaterally, between the left and right limbic systems and in the left parieto-limbic connections). Similar alterations in limbic connectivity have also been reported in patients with schizophrenia [45]. Disruption of the dorsal cingulum bundle in schizophrenia is frequently related to deficits in executive functions and specifically in selective attention [46], [47], aptitudes that are known to be affected in 22q11DS [48], [49], [50].

### Relevance of the Disconnectivity for Other Symptoms Observed in 22q11DS

The abnormal connectivity observed in our study represents the first evidence of disconnectivity in the 22q11DS, as assessed with whole-brain tractography. Similarly to schizophrenia [7], converging evidence points to disconnectivity in 22q11.2 deletion syndrome at several levels. For instance, abnormalities in mismatch negativity were reported using EEG, suggesting disrupted functional fronto-temporal connectivity [51]. Major neuronal disorganization and disturbances in structural neuronal connectivity has been observed in neuropathologic examinations [52]. Finally, exaggerated cortical thinning during adolescence in patients with 22q11DS provides a hint for altered dynamics in the synaptic plasticity of this disorder [53]. All this evidence points to the need to further assess the disconnectivity hypothesis in the context of 22q11DS, e.g. benefiting from the recent advances in the network science [54].

Apart from their potential role in the increased susceptibility to psychosis, the present findings can also be interpreted in the light of other symptoms observed in 22q11DS. Indeed, schizophrenia disorder has received a large interest as it is commonly considered as a behavioral phenotype specific to the syndrome [55]. However, although less specific, other psychiatric diagnoses are even more frequent in patients with 22q11DS. In a large cohort of 172 children, adolescents and adults with 22q11DS, Green and colleagues recently reported a 73% rate of DSM-IV psychiatric diagnoses [56]. The most frequent diagnostic category was anxiety disorder (52%) followed by disruptive disorder (41%) and mood disorder (15%). The significantly decreased connectivity in the limbic system, and more specifically at the level of the cingulum bundles bilaterally, may partly be related to these other psychiatric diagnoses in 22q11DS. Indeed, altered connectivity in the limbic system has been previously reported in several disorders – in depression (reviewed in [57]), ADHD [58] and obsessive-compulsive disorder [59]. Other disorders not specifically associated with 22q11DS also exhibit altered connectivity in the cingulum bundles, such as Alzheimer [60], mild cognitive impairments [61], alcoholism [62], posttraumatic stress disorder [63], underling the potentially non-specific effect of altered limbic connectivity in the development of psychiatric disorders.

Altered connectivity has also been observed in the opposite direction, namely with a relative increase in the number of fibers. Increased connectivity has been located in the frontal and the parietal local (intra-lobar) fibers. In the frontal lobe, increased connectivity coincides with our previous results of increased cortical thickness in children with 22q11DS [53]. It may be the case that the atypically constituted network of frontal long-range connections co-exists with abnormal cortical structure in this region. In 22q11DS adolescents, we observe a collapse in frontal cortical thickness, suggesting that the disorganized cerebral architecture undergoes an uncontrolled pruning, which may later be associated with the onset of schizophrenia. This hypothesis of co-existing connections both at the intra-cortical and the long-range level does not however account for the increased connectivity currently seen in the parietal lobe, as the cortical thickness is not predominantly altered in this region. The causes of the concomitant increased connectivity in the frontal and the parietal lobe may thus rely on another, yet unknown, mechanism.

Except for increased connectivity in the parietal lobe, a notable relationship seems to exist between the direction of the altered connectivity and the gray matter volumetric differences reported in the syndrome, even after correction for the total number of virtual fibers. Indeed, the greater frontal connectivity together with the decreased occipital connectivity parallels the commonly observed rostro-caudal gradient of volumetric changes [16], [64], [65], [66]. The increased connectivity of the intra-parietal connections and the decreased connectivity of the limbic bundles points out the volumetric latero-medial gradient already observed in 22q11DS [66]. As exposed above these “mirrored” findings may be explained by the existence of a strong relationship between the intra-cortical structure and the long-range white matter tract organization.

### Limitations

The current study is the first to provide a whole brain quantification of the three-dimensional axonal tracts in 22q11DS, without the limitations related to the voxel-based analysis. Despite our concern to use one of the most sophisticated techniques available to date, our study bears the same limitations as all other tractography studies. Major limitations, detailed in [34], include among others the current absence of *in vitro* validation of the fiber tracts reconstructed with tractography and the lower resolution of the DTI images compared to T1-weighted images. Recent analysis of tractography reconstruction method revealed bias in accounting for the number of fibers created [67]. The bias, a distance-related effect, will over evaluate the number of fibers in bundles that show a high fractional anisotropy and will under evaluate those with a low FA. Given that to date no accurate correction is available for this bias, we decided to apply a simple linear correction for the length of fiber. This might result in an under evaluation of the number of short connections where FA is low. However, all the MRI images were processed with the same bias correction and therefore no positive results could be accounted for the distance-related effect.

In the present study, we chose to use the Freesurfer parcellation scheme. This choice was driven by the goal to describe the white matter axonal architecture corresponding to regions delimited with primary and secondary sulci, that are the most reliable both at the intra- and inter-subject level. To obtain a more fine grained representation of the white matter changes, we could have used another parcellation with a larger amount of smaller parcels. However, increasing the number of parcel by reducing their size implicates several issues. Firstly, the reliability between subjects will be reduced, introducing supplementary noise in the data. Secondly, a larger number of parcels increases the number of connections to test and therefore raises the severity of the FWE corrections. All these elements would decrease the number of significant findings and would implicate to increase the number of participants for a similar study.

## Supporting Information

Video S1 **360° visualization of the connections within the right frontal lobe.**
(AVI)

Video S2 **360° visualization of the connections within the right parietal lobe.**
(AVI)

Video S3 **360° visualization of the right parieto-occipital connections.**
(AVI)

Video S4 **360° visualization of the connections within the right limbic areas.**
(AVI)

Video S5 **360° visualization of the left fronto-temporal connections.**
(AVI)

Video S6 **360° visualization of the left parieto-limbic connections.**
(AVI)

Video S7 **360° visualization of the connections within the left limbic areas.**
(AVI)

Video S8 **360° visualization of the connections within the left occipital lobe.**
(AVI)

Video S9 **360° visualization of the inter limbic connections.**
(AVI)
